# Establishment and Molecular Cytogenetic Characterization of a Cell Culture Model of Head and Neck Squamous Cell Carcinoma (HNSCC)

**DOI:** 10.3390/genes1030388

**Published:** 2010-11-11

**Authors:** Verena L. Bauer, Ludwig Hieber, Quirin Schaeffner, Johannes Weber, Herbert Braselmann, Reinhard Huber, Axel Walch, Horst Zitzelsberger

**Affiliations:** 1 Department of Radiation Cytogenetics, Helmholtz Zentrum München, German Research Center for Environmental Health, Ingolstädter Landstr.1, 85764 Neuherberg, Germany; E-Mails: Verena.bauer@helmholtz-muenchen.de (V.L.B.); ludwig.hieber@helmholtz-muenchen.de (L.H.); quirin.schaeffner@helmholtz-muenchen.de (Q.S.); joh.weber@gmail.com (J.W.); braselm@helmholtz-muenchen.de (H.B.); rhuber@helmholtz-muenchen.de (R.H.); 2 Institute of Pathology, Helmholtz Zentrum München, German Research Center for Environmental Health, Ingolstädter Landstr.1, 85764 Neuherberg, Germany; E-Mail: axel.walch@helmholtz-muenchen.de

**Keywords:** HNSCC, CAL 33, cell lines, cytogenetics

## Abstract

Cytogenetic analysis of head and neck squamous cell carcinoma (HNSCC) established several biomarkers that have been correlated to clinical parameters during the past years. Adequate cell culture model systems are required for functional studies investigating those potential prognostic markers in HNSCC. We have used a cell line, CAL 33, for the establishment of a cell culture model in order to perform functional analyses of interesting candidate genes and proteins. The cell line was cytogenetically characterized using array CGH, spectral karyotyping (SKY) and fluorescence *in situ* hybridization (FISH). As a starting point for the investigation of genetic markers predicting radiosensitivity in tumor cells, irradiation experiments were carried out and radiation responses of CAL 33 have been determined. Radiosensitivity of CAL 33 cells was intermediate when compared to published data on tumor cell lines.

## 1. Introduction

Head and neck squamous cell carcinomas (HNSCC) are one of the most frequent cancer types worldwide with more than 900,000 people being affected each year [1]. An estimated 35,500 new cases and 7,800 deaths are expected for 2010 in the U.S. [2] and therewith HNSCC represent a major cause for morbidity and mortality. Cancers of the head and neck include malignancies of different anatomical locations of the head and neck region (*i.e.*, oral cavity, lips, tongue, gingiva, salivary gland, larynx, pharynx, middle ear and nasal cavity). In general linguistic usage, the term “head and neck cancer” describes squamous cell carcinoma of the most affected areas: the oral cavity, larynx and pharynx [3]. Despite ongoing developments in tumor therapy, survival rates and overall prognosis of patients suffering from HNSCC have not improved significantly during the last 30 years [4], with 5-year and 10-year relative survival rates not exceeding 50% and 61%, respectively [5]. Classical prognostic factors of HNSCC have been established concerning general parameters like tumor staging and histological grading, which unfortunately frequently fail to predict biological tumor behavior and patient survival [1,6]. As a consequence, tumor therapies still fail leading to locoregional recurrence, metastasis and/or second primary tumors [7]. HNSCC development and progression occurs via accumulating genetic alterations that can cause an activation of proto-oncogenes or an inactivation of tumor-suppressor genes. Therefore, it is of special interest to investigate the molecular mechanisms of HNSCC and to identify new potential molecular biomarkers. For that reason, many studies investigate HNSCC tumor tissues using different experimental approaches and analyzing diverse endpoints like copy number alterations and gene-expression changes [8,9]. Resulting potential molecular markers and candidate genes must be validated on primary tumor tissues [10,11] before functional studies are accomplished elucidating their diagnostic/prognostic properties. Well characterized cell lines are required for the establishment of reliable cell culture systems and many HNSCC cell lines have been an invaluable tool for researchers investigating various aspects of HNSCC [12]. Particularly, studies analyzing the diverging responses of HNSCC tumor cells to therapeutic approaches are of special interest, since it is still unknown why tumors expressing similar phenotypes respond differently to therapies [6]. CAL 33 is a HNSCC cell line that was established by Gioanni *et al.* [13] from a biopsy extracted prior to therapy from a moderately differentiated squamous cell carcinoma of the tongue of a 69-year old male. Various studies have used CAL 33 cells as a model system for the functional testing of therapeutics and for the analyses of molecular markers [14,15,16,17,18,19]. The aim of this study was the establishment of a functional cell culture system based on the HNSCC cell line CAL 33 in order to investigate potential candidate genes playing a role in radiation sensitivity with respect to their diagnostic or prognostic properties. This report focuses on the molecular cytogenetic characterization of the original cell line that has been performed prior to genetic engineering and functional analyses.

## 2. Results and Discussion

CAL 33 is a widely used head and neck squamous cell cancer (HNSCC) cell line for testing of therapeutic agents [15,16,17,18,19] and investigating molecular markers of HNSCC [14]. A further potential application is to perform functional studies on specifically genetically-engineered clones of CAL 33. The veracity of experimental results obtained from cell culture models are based upon the correct derivation of the cell lines. A useful tool to determine the cell line derivation, the evolutionary development of the cell line in culture and changes that are caused by genetic engineering is a detailed molecular cytogenetic characterization. Therefore, various molecular cytogenetic approaches were performed in order to investigate karyotypic changes in the head and neck cancer cell line CAL 33 and in derived cell clones after gene transfection. The results obtained from Spectral Karyotyping (SKY), array comparative genomic hybridization (array CGH) and fluorescence *in situ* hybridization (FISH) are summarized in Table 1.

### 2.1. Cytogenetic Characterization of CAL 33 Cells and Derived Cell Clones After Gene Transfection

#### 2.1.1. Structural Rearrangements Detected by Spectral Karyotyping (SKY)

**Figure 1:**
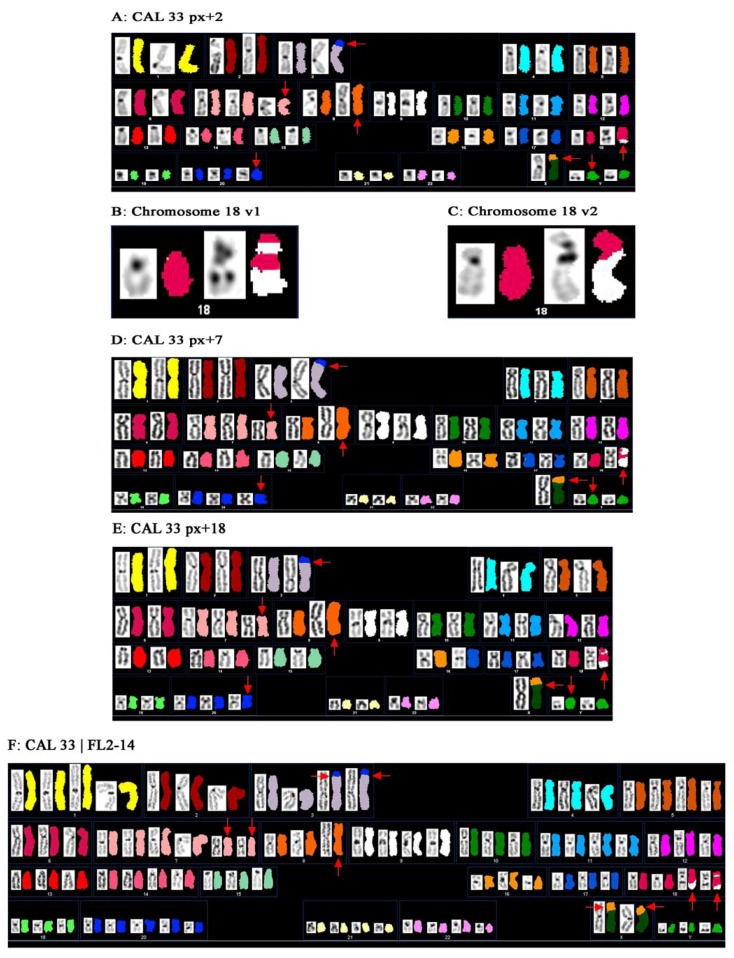
SKY analysis of the HNSCC cell line CAL 33. Homologous chromosomes appear in distinct colors and DAPI banding. Chromosomal rearrangements are detected by color junctions that are pointed out by arrows. (**A**) SKY ideogram of CAL 33 passage x + 2 (px + 2). Translocations involving chromosomes 3, 9, 16, 18, 20 and X can be observed besides two isochromosomes (i(7p) and i(8q)). Moreover, two additional chromosomes (chromosomes 20 and Y) are present. All inter-chromosomal aberrations are described with the karyotype: 49,YY,der(X)t(X;16)(p22;?),der(3)t(3;20)(p25;?),i(7)(p10),i(8)(q10),der(18)t(18;9)(p13;?)t(18;9)(q21;?),+7,+20; (**B**) Derivative chromosome involving chromosomes 18 and 9, variant 1 (v1): der(18)t(18;9)(p13;?)t(18;9)(q21;?); (**C**) Derivative chromosome involving chromosomes 18 and 9, variant 2 (v2): der(18)t(18;9)(p10;q10); (**D**) SKY ideogram of CAL 33 passage x + 7 (px + 7). The karyotype complies with the karyotype described in (A); (**E**) SKY ideogram of CAL 33 passage x + 18 (px + 18). The karyotype complies with that described in (A) and (B); (**F**) SKY karyogram of the transfected cell line CAL 33/FL2-14. This cell line mainly mirrors the karyotype of the initial cell line CAL 33 (Figures A, D and E) but shows a nearly tetraploid chromosome set.

Numerical and structural rearrangements were analyzed by SKY, a widely used cytogenetic method visualizing all 24 human chromosomes in different colors within a single experimental approach by applying “Whole Chromosome Paint”-(WCP)-probes labeled with a different combination of fluorescent dyes [20]. SKY analysis of the cell line CAL 33 detected rearrangements involving chromosomes 3, 7, 8, 9, 16, 18, 20 and X, and additional chromosomal material could be identified for chromosomes 7, 20 and Y. The resulting karyotype for the investigated cell line CAL 33 is shown in Figure 1A and Table 1 and described as: 49,Y,Y,der(X)t(X;16)(p22;?),der(3)t(3;20)(p25;?),i(7)(p10),i(8)(q10),der(18)t(18;9)(p13;?)t(18;9)(q21;?),+7,+20.

For the rearrangement involving chromosomes 9 and 18, two different cytogenetic variants were observed indicating different sub-clones in CAL 33 cells. One marker chromosome 18 showed material from chromosome 9 on both the p- and q-arm (variant 1, Figure 1B), while the other marker chromosome 18 only displayed material from chromosome 9 on the q-arm (variant 2, Figure 1C). Out of 16 analyzed metaphases, eleven (69%) showed variant 1 and five (31%) showed variant 2. Gioanni *et al.* [13] reported for the first time on the establishment and characterization of the CAL 33 cell line. Karyotyping of the primary culture at passage 10, which was very close to the original tumor by G-banding revealed a moderate hyperploidy, with an average number of 49 chromosomes per cell. They detected several marker chromosomes described as 3p+, i(7q), Xp+, i(7p) and one unidentified marker chromosome. After applying SKY analysis we succeeded in determining the karyotype in more detail and in specifying marker chromosomes (Figure 1, Table 1). The marker chromosomes i(7p), 3p+, Xp+, 9p+ and der(9)?? described by Gioanni *et al.* [13], as well as the mean number of chromosomes per cell (49) were confirmed by our studies. Chromosomes 3p+, Xp+ and der(9)?? were specified as der(3)t(3;20)(p25;?), der(X)t(X;16)(p22;?) and der(18)t(18;9)(p13;?)t(18,9)(q21;?) or der(18)t(18;9)(p10;q10), respectively. The initially indicated isochromosome 7q must have been misclassified since we detected an isochromosome 8q by SKY instead of i(7q). In addition, we could clarify the unknown marker chromosome mentioned by Gioanni *et al.* [13] that could be assigned as the additional chromosome 20. Although in the initial publication an additional chromosome Y was not mentioned, we found this in almost every metaphase of CAL 33 cells. The reason for this discrepancy could be either that we are facing a karyotypic evolution of the cell line since its establishment in 1988, or that Gioanni *et al.* [13] analyzed incomplete metaphases. Many publications have reported on the phenomenon of karyotypic evolution in cell culture [21,22,23,24]. Bahia *et al.* [25] and Watson *et al.* [26], for instance, investigated independently cultured strains of breast cancer cell lines and noticed, besides several common alterations, a significant number of additional and unique aberrations that emerged during *in vitro* cultivation and genotypic evolution. As a consequence of genetic alterations, phenotypic characteristics of individual cell lines can be altered and lead to different and inconsistent results after functional testing of *i.e.*, therapeutic agents. Therefore, we analyzed the CAL 33 cell line at different passage numbers, in order to check chromosomal stability during *in vitro* culturing. As depicted in Figure 1, the rearrangements detected by SKY were the same for passage x + 2 (Figure 1A), x + 7 (Figure 1D) and x + 18 (Figure 1E), indicating a stable karyotype for CAL 33 cells in culture. Moreover, the detected alterations are in good agreement to those published for the primary culture at passage number 10 [13], which shows that the genetic status of CAL 33 still mirrors that of the original tumor—an important criterion for all cell lines that will be used for the establishment of a cell culture model. The Fanconi Anemia (FA)/BRCA-pathway is involved in DNA replication and controls homology-directed DNA repair [27,28]. Alterations of components of this pathway have been observed in HNSCC and proposed to be of interest for clinical implications [29]. The gene *FANCA* has been found amplified in a cohort of HNSCC exclusively treated by radiation therapy and correlated negatively with patients’ survival time after therapy [11], suggesting a role for FANCA expression in radiosensitivity of tumor cells. In order to investigate the influence of FANCA expression on the radiosensitivity of HNSCC tumor cells, we stably transfected CAL 33 cells with a FANCA-overexpressing vector. One of the resulting clones, CAL33/FL2-14, was karyotyped by SKY and revealed a karyotype similar to the original cell line CAL 33 (Figure 1F). However, it is of special interest that these cells were almost tetraploid, in contrast to the diploid original cell line. This could either be a consequence of genetic manipulation itself, or a result of FANCA-overexpression indicating an involvement of this gene in genetic stability. 

Overall, the findings revealed by SKY analysis of CAL 33 cells are in good concordance with Squire *et al.* [30], who also reported rearrangements of the above mentioned chromosomes in a large number of HNSCC cell lines. Previous analyses have shown that SKY analysis is a very useful tool to uncover the very complex karyotypes of HNSCC [30,31,32]. However, for SKY analysis the availability of sample metaphase spreads is crucial—a limiting factor for the investigation of all solid tumors. Experimental approaches are restricted to established or primary cell lines and cannot be accomplished using solid tumor material. Moreover, although gross numerical changes are revealed by SKY analyses, smaller copy number alterations are not detectable. Therefore, other molecular methods should be used to complement SKY analyses in order to identify copy number changes at higher resolution.

**Table 1:** Cytogenetic analyses of CAL 33 cell lines.

**Cell Line**	Chromosomal Changes Detected by SKY	Copy Number Changes Detected by array CGH		Validation by FISH
		DNA Gains	DNA Losses	
**CAL 33**	Px + 2: 49,Y,Y,der(X)t(X;16)(p22;?), der(3)t(3;20)(p25;?), i(7)(p10),i(8)(q10), der(18)t(18;9)(p13;?)t(18;9)(q21;?),+7,+20	Px + 2: 3q, 7p, 8q, 9p24.3-22.2, 9p13.2-p11.1, 9q, 16p13.3-11.2, 20	Px + 2: 3p, 4q34.3-q35.1, 8p, 12q24.3, 16q22.1, 18q12.2-23	der(3)t(3p;20q); i(3q); i(7p); i(8q); der(X)t(Xp;16p); -4q35.1; -12q24.31;
	Px + 7: 49,YY,der(X)t(X;16)(p22;?), der(3)t(3;20)(p25;?),i(7)(p10),i(8)(q10), der(18)t(18;9)(p13;?)t(18;9)(q21;?),+7,+20	Px + 13: 1p36.3-p34.1, 1p13.3-q23.3, 1q25.2-q25.3, 1q32.1, 3q, 7p, 7q11.2, 7q22.1, 8q, 9p24.3-p22.2, 9p13.3-p11.1, 9q, 11p15.5-p15.2, 11p11.2, 11q12.1-q13.5, 11q23.3-q24.2, 12q13.1-q14.1, 16p13.3-p11.2, 17p, 17q11.2-21.3, 17q22-25.3, 19p13.3-13.1, 19q13.1-q13.4, 20, 22q	Px + 13: 2p16.3-p16.2, 3p, 4q34.3-q35.1, 8p, 12q24.3, 16q21, 16q22.1,17q21.3-q22, 18q12.2-q22, 18q22-q23	-
	Px + 18: 49,YY,der(X)t(X;16)(p22;?), der(3)t(3;20)(p25;?),i(7)(p10),i(8)(q10), der(18)t(18;9)(p13;?)t(18;9)(q21;?),+7,+20	Px + 24: 1p36.3-p34.1, 1p13.3-q23.1, 1q32.1-32.2, 3q, 7p, 8q12.1-q13.1, 9p23-p22.2, 9p12-p11.1, 9q, 12q13.1-q14.1, 16p13.3-p11.2, 19p13.3-13.1, 19q13.1-q13.4, 20, 22q	Px + 24: 3p, 8p, 12q24.3, 17q21.3-q22, 18q11.2-q21.3, 18q22.1-q23	-
**CAL 33/ FL2-14**	85,YYY,2x der(X)t(X;16)(p22;?), +2x der(3)t(3;20)(p25;?),+2x i(7)(p10),+i(8)(q10), +2x der(18)t(18;9)(p13;?)t(18;9)(q21;?),+1,+1,+2,+4, +5,+5,+6,+7,+7,+9,+9,+10,+11,+11,+12,+13,+14,+14,+15,+16,+17,+19,+19,+20,+20,+20,+21,+21,+22,+22	3q, 7p, 8q, 9p24.3-22.2, 9p13-p11.1, 9q13-q22, 9q31, 9q34, 14q, 16p13.3-p11.2, 17p13-p12, 20	2, 3p, 4q34.3-q35.1, 8p, 12q24.3, 15q, 16q22, 17q21-q25, 18q12-q22, 18q22-q23	-

Px + ...= passage number x+…; FL2-14 = transfected clone.

#### 2.1.2. Copy Number Alterations Detected by Array CGH

**Figure 2:**
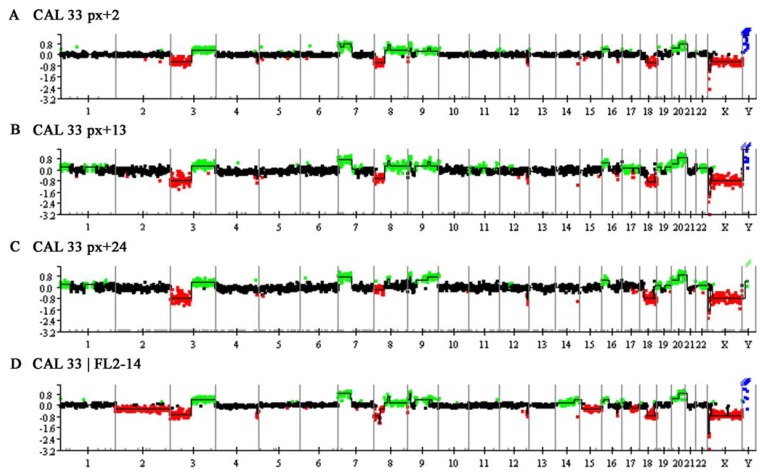
Whole genomic array CGH profile of the HNSCC cell line CAL 33. Along the X-axis, which represents the individual chromosomes and chromosome number, log2-ratios for ever single BAC clone are depicted on the Y-axis. Normal regions are shown in black, regions of DNA gain are displayed in green and regions of DNA loss in red. Amplifications are represented in blue. Since sex-mismatched hybridizations were performed, alterations on chromosomes X and Y were not evaluated. For detailed descriptions of altered regions see Table 1. (**A**) Array CGH of CAL 33, passage x + 2 (px + 2). DNA gains are detected on chromosomes 3q, 7p, 8, 9, 16p and 20. Deletions are seen on chromosomes 3p, 4q, 8p, 9p, 12q, 16q and 18q; (**B**) Array CGH of CAL 33, passage x + 13 (px + 13). This passage shows some additional copy number alterations compared to alterations described in (A). These are DNA gains on chromosome 1, 7q, 11, 12q, 17, 19 and 22. Additional DNA losses involve chromosomes 2 and 17; (**C**) Array CGH of CAL 33, passage x + 24 (px + 24). The highest passage analyzed appears very similar to passage 13, however, DNA gains on chromosomes 8q and 17 and the deletion on chromosome 2 are not detected; (**D**) Array CGH of the transfected cell line CAL 33/FL2-14. The transfected cell line still shows the main genomic characteristics of the initial, non transfected cell line of passage x + 13 (px + 13, B). Additional alterations are DNA gains on chromosome 14 and deletions on chromosomes 2 and 15.

Comparative genomic hybridization (CGH) [33] and advanced array-based CGH (array CGH) [34] allow the investigation of chromosomal imbalances (*i.e.*, DNA gains, DNA losses and amplifications) of tumor genomes, originating either from fresh, frozen, or formalin-fixed and paraffin embedded (FFPE) tissues or from cell lines. Array CGH of CAL 33 using BAC-arrays of 1 MB resolution [35] revealed seven gains on chromosomes 3, 7, 8, 9, 16 and 20, and seven losses on chromosomes 3, 4, 8, 9, 12, 16 and 18 that are shown in Figure 2A and summarized in Table 1. These findings are in good concordance to CGH data of 114 primary HNSCC cases analyzed by Bauer *et al.* [11] as well as to other published data from HNSCC available on www.progenetix.net [36]. Thus, our copy number data from CAL 33 reflect some of the most typical copy number changes in HNSCC, like chromosomal imbalances on chromosomes 3p/q, 4q, 8q, 9p, 16q and 18q (for review see [37]) and represent a convincing proof for the derivation of the CAL 33 cell line from HNSCC. 

Similar to SKY analysis, we performed array CGH experiments with different passages of CAL 33 cells in order to investigate karyotype evolution in cultured cells (Figure 2 and Table 1). Most of the alterations detected in passage x + 2 were also found at higher passages. These were DNA gains on chromosomes 3q, 7p, 9, 16p and 20 and DNA losses on chromosomes 3p, 4q, 8p, 12q, and 18. Interestingly, there were two regions of DNA loss on chromosome 18q separated by a region of normal copy number in passages x + 13 and x + 24 (Table 1), while in passage x + 2 only one large deletion was detected. DNA gain of 8q was observed in passages x + 2 and x + 13, while the gained region in passage x + 24 was smaller and did not affect the whole chromosome arm. In contrast to passage x + 2, array CGH of passages x + 13 and x + 24 revealed identical DNA gains on chromosomes 1p, 12q, 19 and 22 and DNA losses on chromosome 17q. Both passages also showed DNA gains on chromosome 1q, although the altered regions varied. Passage x + 13 showed a couple of unique alterations like DNA gains on 7q, 11 and 17 and deletions on 2p and 16q, that were not detected in passage x + 2 and x + 24. These results again point to a cytogenetic evolution in CAL 33 cells in culture which results in slightly different genomic profiles when analyzed by array CGH. Array CGH results of the transfected cell line CAL 33/FL2-14 (Figure 2D) comply best with the copy number alterations detected in passage x + 13. This could be due to the fact that genetic engineering of the original cell line was performed at passage x + 7, which is close to the investigated passage number x + 13. Common alterations between CAL 33 at passage x + 13 and the transfected cell line were DNA gains on chromosomes 3q, 7p, 8q, 9, 16p, 17p and 20 and DNA losses on chromosomes 3p, 4q, 8p, 12q, 16q and 18q. Gains observed on 1, 7q, 11, 12q and 17q for passage x + 13 of CAL 33 cells could not be detected in the transfected cell line indicating that they might not be important for the tumor phenotype of CAL 33 cells. CAL 33/FL2-14 showed an additional DNA gain on chromosome 14 and additional deletions of chromosome 2, 15q and 17q that point to genomic instability induced by the gene transfection.

#### 2.1.3. Fine-Mapping by FISH and Complementary Data Analysis

**Figure 3:**
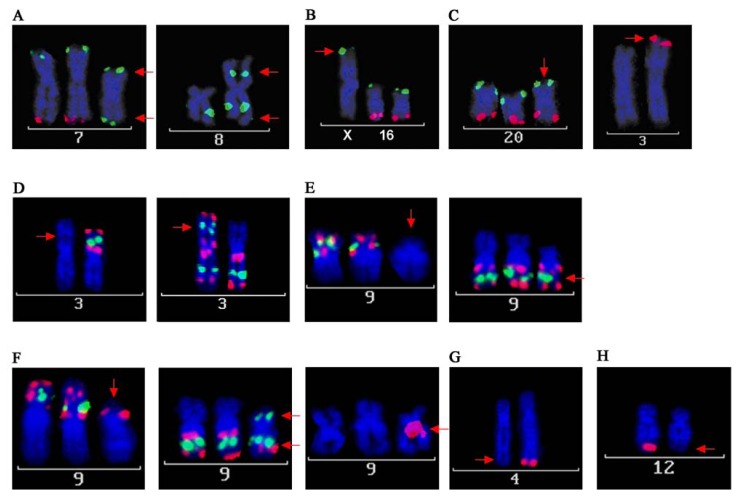
FISH analyses of CAL 33. BACs were labeled with biotin (green) or digoxigenin (red) and hybridized on metaphase spreads. (**A**) Confirmation of isochromosome formation of chromosomes 7p and 8q. For i(7p) BAC clones mapping to the terminal ends of 7p (green) and 7q (red) were hybridized. i(7p) is indicated by green signals on both chromosome arms. For i(8q) a biotin-labeled (green) BAC clone mapping to 8q was used. i(8) can be detected as a metacentric chromosome, displaying 8q-signals on both chromosome arms; (**B**) BAC clones mapping the terminal ends of chromosome 16p (green) and 16q (red) were hybridized. Green signals on the terminal part of chromosome X indicate that the translocated material from chromosome 16 derives from chromosome 16p; (**C**) Trisomy of chromosome 20 is clearly detectable with BAC clones hybridizing on 20p (green) and 20q (red). Red signals on chromosome 3p indicate, that the translocated material derives from chromosome 20q; (**D**) Confirmation of isochromosome 3q: hybridizing three BAC clones mapping to 3p (red, green and red) depicts one single p-arm. In a separate hybridization, three BAC clones mapping to 3q (red, green and red) revealed i(3q); (**E**) Characterization of marker chromosome der(18)t(18;9)(p10;q10), variant 2. Similar to (D), three different BAC clones mapping to 9p and 9q were used (red, green and red). Hybridization of only the 9q-BAC clones shows that the marker chromosome contains material deriving from 9q, but not from 9p; (**F**) Characterization of marker chromosome der(18)t(18;9)(p13;?)(q21;?), variant 1. Applied BAC clones for chromosome 9 were equal to (E). A BAC clone mapping close to the centromere of chromosome 18 is displayed in red. This marker chromosome shows a more complex pattern of signals. BACs from 9p as well as from 9q hybridized on its p-arm and only two of the signals of the three BAC clones from 9q can be detected on its q-arm. The centromeric part represents chromosome 18; (**G**) Confirmation of DNA loss on chromosome 4q. The red signal of a BAC clone mapping to that area can be detected only in one of the homologous chromosomes; (**H**) Confirmation of DNA loss on chromosome 12q. The red signal of a BAC clone mapping that area can be detected only in one of the homologous chromosomes.

Both chromosomal rearrangements and copy number alterations can point to interesting candidate genes that may play a role in tumor development and/or progression. In order to narrow down breakpoints of chromosomal rearrangements, SKY analyses are often followed by positional cloning experiments using FISH based approaches with yeast or bacterial artificial chromosomes (YAC/BAC). The chromosomal breakpoints can affect genes that may be disrupted and thereby either can cause loss of function or translocation to another part of the genome leading to altered gene function [38,39]. On the other hand, copy number alterations detected by array CGH analyses of tumor genomes are often confirmed by FISH on tumor tissue sections, allowing a validation of specific cases or to validate specific genetic imbalances in larger tumor cohorts, *i.e.*, using tissue arrays [11,40,41]. In this study, FISH experiments were performed on metaphase spreads of the cell line CAL 33 in order to further elucidate specific aberrations detected by array CGH and SKY analyses. Using different cytogenetic approaches for a comprehensive and complementary analysis, complex karyotypes can be uncovered in more detail. The results of array CGH for CAL 33 closely match the described karyotype determined by SKY analysis. Isochromosomes 7p and 8q are clearly verified by DNA gains of 7p and 8q in array CGH, respectively. Loss of 8p detected by array CGH reflects the fact that i(8q) substitutes one of the homologous chromosomes, while normal copy number for 7q in array CGH confirms the additional existence of i(7p) in addition to the two homologs of chromosomes 7. This was also shown by FISH on metaphase spreads of CAL 33 cells (Figure 3A) using BAC clones mapping to chromosomes 7 and 8 (Table 2). i(8q) formation and 8q gains are some of the most common structural and copy number alterations in HNSCC and have been previously reported many times in various studies [1,42,43,44,45]. i(7p) occurrence is much less frequently reported in the literature [46], however, the related gain of 7p has again been found in a number of studies from Huang *et al.* [47], Freier *et al.* [1], and Patmore *et al.* [37]. This can be explained by the fact that structural rearrangements like isochromosomes can only be detected from analyzing metaphase preparations. These cannot be obtained from archived tumor tissues, however, these tissues are mainly used to investigate genomic changes and this can lead to a bias in the detection of 7p isochromosomes and 7p gains. The DNA gain on chromosome 16p showed that the translocation in CAL 33 cells from chromosome 16p on chromosome Xp (der(X)t(Xp;16)) is unbalanced, which was confirmed by FISH (Figure 3B). SKY, as well as FISH and array CGH, revealed a trisomy of chromosome 20 (Figure 2A and Figure 3C). FISH analysis demonstrated that the translocated part of chromosome 20 on chromosome 3 derives from 20q and not 20p (Figure 3C). This part of 20q is obviously present in addition to trisomy 20, as indicated by a higher gain of the distal part of 20q in array CGH and FISH. Copy number gains for chromosome 20 are again one of the most common alterations in HNSCC, that are detected both in HNSCC cell lines [1,45,48] and in primary HNSCC [8,11,47]. The additional chromosome Y detected by SKY and indicated by an amplification of the whole chromosome in array CGH is more likely to be a side effect of aneuploidy occurring very frequently in HNSCC [49,50] rather than being a tumor-specific alteration.

Besides the concordant alterations between SKY and array CGH analyses, there were a few inconsistencies detected with both techniques. Complementary analysis using FISH with BAC clones allowed us to elucidate these discrepancies. Chromosome 3 showed a DNA loss and gain for the entire p- and q-arm. FISH using BAC clones mapping either the short or the long chromosome arm (Table 2 and Figure 3D) identified one of the chromosomes 3 as i(3q). However, this rearrangement could not be detected by SKY since the DAPI banding pattern was not informative. i(3q) and loss of 3p/gain of 3q are among the most important structural and numerical alterations in HNSCC and were reported in almost every cytogenetic study on these tumors and are the most frequently reported finding [1,11,37,51]. These alterations have also been suggested to be early events in tumor development and to play a role in HNSCC tumor progression [46,52]. 

Another discrepancy was noticed for large DNA gains on chromosome 9p/q in array CGH. SKY analysis revealed two marker chromosomes involving chromosomes 18 and 9 (variants 1 and 2, Figure 1B and C). FISH experiments with BAC clones either mapping chromosome 9p or 9q uncovered chromosome 9 as a very unstable chromosome in this cell line. Three different variants could be observed on one metaphase preparation possibly indicating different sub-clones of CAL 33. First, a marker chromosome showing the 9q FISH pattern but no 9p signal was observed (Figure 3E). This FISH pattern reflects the marker chromosome der(18)(18;9) variant 2 as described by SKY analysis. The other marker chromosome (variant 1) displayed the distal pattern of chromosome 9q on its q arm and the centric part of chromosome 9q on its p-arm, which is depicted in Figure 3F. Additionally, a signal of chromosome 9p appears on its p-arm, which suggests that this rearrangement involves an inversion on chromosome 9. To further clarify all chromosome 9 alterations in CAL 33 cells, additional experiments (e.g., positional cloning, array-paining) are needed. The maximum molecular resolution of SKY is at about 3 MB [53] and therefore may not detect the smaller alterations detected by BAC-FISH. Chromosome 9 alterations again have been reported in many studies, including studies on the molecular evolution and progression of HNSCC. Califano *et al.* [52] suggest loss of 9p as one of the initial events in progression from normal squamous epithelium to dysplasia and carcinoma. FISH analysis with the resolution of BAC clones opens up the potential to identify key candidate genes. Interesting candidate genes like CDKN2B on 9p21 are being discussed to function in critical pathways in HNSCC tumor development and progression [54]. 

**Table 2:** BAC clones for FISH on CAL 33 cells.

BAC clone	Chromosome	Localization (bp)*	FISH Labeling
RP11-238A9	3p26.1	4516514-4676956	Digoxigenin
RP11-424L2	3p22.1	41012714-41179560	Biotin
RP11-220O14	3p12.3	77503140-77655193	Digoxigenin
RP11-114I8	3q12.2	99997014-100148683	Digoxigenin
RP11-30J14	3q25.1	151663244-151830918	Biotin
RP11-392H18	3q28	190013809-190173313	Digoxigenin
RP11-228f3	4q35.1	185636344-185802231	Digoxigenin
RP11-20N02	7p22.3	758897-908887	Biotin
RP11-664B05	7q36.3	158712708-158902209	Digoxigenin
RP11-627A06	8q21.3	90721901-90763832	Biotin
RP11-509J21	9p24.2	3544199-3705631	Digoxigenin
RP11-33K8	9p21.3	24101721-24252438	Biotin
RP11-614P24	9p13.2	37044198-37212970	Digoxigenin
RP11-265B8	9q21.11	71568181-71737561	Digoxigenin
RP11-23B15	9q22.33	100545008-100703779	Biotin
RP11-269P11	9q33.3	128193295-128367188	Digoxigenin
RP11-158L12	12q24.31	125491151-125667388	Digoxigenin
RP11-22E02	16p13.13	12342021-12505232	Biotin
RP11-466E19	16q23.1	77319681-77480278	Digoxigenin
RP11-1076F02	18q11.2	19542825-19733692	Digoxigenin
RP11-640A09	20p13	113369-267637	Biotin
RP11-631L08	20q13.33	62410553-62589484	Digoxigenin

* Localization according to ensemble release 58 (www.ensembl.com).

Further, small deletions on chromosomes 4q34-35 and 12q24.3 were observed in the array CGH profile of CAL 33 (Figure 2A). These small deletions were obviously not detectable by SKY analysis but were verified by FISH (Figure 3G and H). The BAC clone used for the evaluation of the 4q deletion (RP11-228F3) maps an interesting gene, MLF1P. This potential candidate gene plays an important role in centromere assembly and is critical for correct chromosome segregation during mitosis [55,56]. Loss of function as a consequence of chromosomal deletion could be relevant for the well known effect of polyploidy in HNSCC. Another interesting candidate gene maps on a BAC clone from the deleted region on chromosome 12q (RP11-158L12). BRI3BP is known to be involved in apoptosis [57] and most interestingly in drug-induced apoptosis. Yamazaki *et al.* [58] state that reduction or loss of BRI3BP might result in inefficient elimination of harmful cells, favoring tumor development. Their finding that overexpression of this gene enhances drug-induced apoptosis makes it an interesting target for medical treatments. Experiments with cell lines like CAL 33 could elucidate the relevance of BRI3BP as a pharmacological target in HNSCC. 

Before establishing cell culture models for functional testing, the above mentioned cytogenetic methods provide adequate tools to check the derivation and genetic features of cell lines. This is also very important, as it is not uncommon that cell lines get contaminated during culture. In 2008, a review published by Lacroix *et al.* [59] reported the persistent use of “false” cell lines in worldwide research. This scenario can be caused by two major events: Firstly, the establishment of new cell lines from e.g., tissues by culturing the wrong cell type, and secondly by cross-contamination of existing cell lines, frequently with robust and rapidly multiplying cells like HeLa cells, that over grow the original cell culture [60]. Schweppe *et al.* [61] screened 40 thyroid cell lines and only about half of them turned out to have unique genetic profiles. Facing this problem, it is especially important to test newly established and acquired cell lines, in order to evaluate and approve their identity and derivation. Liebertz *et al.* [48] established a novel HNSCC cell line (USC-HN1) and characterized the obtained cells in detail by cloning, immunohistochemistry (IHC), cytogenetic analysis and viral screenings. IHC experiments using tissue-specific and typical antibodies can quickly give information about cell origin. Cytokeratine staining in IHC can be used to prove the epithelial origin of cell lines, since these proteins are expressed in epithelial tissues and used as epithelial markers [62]. Using this method, we could allocate CAL 33 cells as epithelial cells [63] (data not shown) and confirm the immunochemical studies by Gioanni *et al.* [13]. The cytokeratine staining of CAL 33 cells was clearly positive when compared to fibroblasts (control cells).

### 2.2. Establishment of a CAL 33 Cell Culture Model for Functional Studies of Radiosensitivity

**Figure 4:**
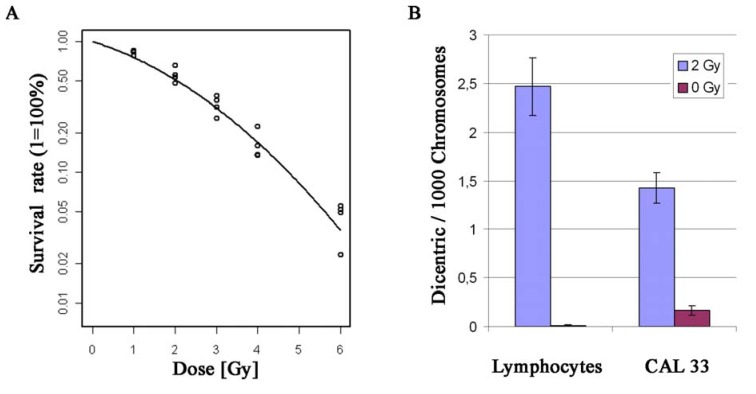
Radiation experiments with CAL 33 cells. (**A**) Survival curve of CAL 33 after different doses of γ-irradiation (1, 2, 3, 4 and 6 Gy); (**B**) Rate of dicentric chromosomes in CAL 33 cells and normal human lymphocytes after 2 Gy γ-irradiation and in corresponding control cells (0 Gy). Error bars represent the standard error of the mean.

*In vitro* model systems are very common in tumor studies as they enable researchers to investigate phenotype characteristics which are dependent on the genetic composition of the cells. The generation of several cell lines from premalignant lesions as well as from primary carcinomas and metastatic deposits led to the establishment of experimental models for multistep cancer progression in HNSCC [12,52,64]. Moreover, cell line systems are widely used to identify the molecular characteristics of different cancer types, not only HNSCC. Neve *et al.* [65] described recurrent genomic and transcriptional characteristics of 51 breast cancer cell lines. An important finding here was that the molecular genetic composition of the analyzed breast cell lines mirrors that of primary tumors. This is an essential issue, since cell line model systems are widely used to model the *in vivo* situation. In our study, the derivation of CAL 33 cells from HNSCC was confirmed by similar alteration patterns observed in the cell line and HNSCC tumor genomes, as discussed in the previous section. A major focus of HNSCC research is the identification of diagnostic biomarkers and prognostic markers that are expected to play an important role in tumor classification and behavior as well as in treatment response of tumors. Classical clinical staging parameters of the Union International Contre le Cancer (UICC; [66]), like patient-related (e.g., age, gender) and histomorphological factors (e.g., resection status, grading), are considered as established prognostic factors. Nonetheless, it still remains widely unclear, why histopathologically similar tumors behave differently with respect to tumor progression and/or response to therapies [6]. Since HNSCC development occurs as a consequence of accumulating genetic alterations, special attention is drawn to the identification of molecular mechanisms and genetic prognostic factors. A variety of promising factors have been identified so far. Human Papilloma Virus (HPV) is one of the most intensively studied biomarkers and has been reported to play an important role in tumor development in a subgroup of HNSCC providing diagnostic, prognostic and therapeutic options [7,67]. Various genes have been discovered in different studies and represent promising biomarkers (for review see [68]). The function of these genes, including *CDKN2A*, *TP53*, *PTEN*, *CCND1*, *EGFR*, *VEGF*, *COX-2*, can be altered by different molecular alterations like DNA gains and losses, structural rearrangements, epigenetic events, gene dosage independent modifications of expression or viral gene deactivation. Even though these promising prognostic molecular markers have been identified so far, none of them was validated as a reliable biomarker for diagnosis and prognosis. Additionally, Chang and Califano [7] suggested that a good biomarker should predict response to applied cancer therapies and moreover to be a therapeutic target itself. Functional studies with HNSCC cell lines can be used to investigate the effect of potential therapeutics, as well as the effect of certain induced alterations on the cells’ response to therapeutics [65,69,70,71]. Radiotherapy represents one of the central therapy options for HNSCC. Consequently, an important issue is to investigate the individual radiosensitivity of HNSCC, which could lead to diverging response to radiotherapy of different cases. Radiation experiments on HNSCC cell lines therefore represent an important issue of therapeutic research, either investigating overall radiation sensitivity [72] or performing functional studies in order to analyze the effect of certain genes/proteins on cellular radiosensitivity [73]. As already mentioned in the previous section, we transfected the cell line CAL 33 with an FANCA overexpressing vector in order to establish a cell culture model for the investigation of the effect of FANCA-overexpression on radiosensitivity. The resulting cell lines, like CAL 33/FL2-14, are currently characterized for radiation response. In order to get information on the radiosensitivity of the original CAL 33 cells, we performed radiation experiments by using the colony forming ability assay after exposure to different doses of Cs^137^ γ-irradiation. The resulting survival curves describe the growth behavior after radiation exposure. Moreover, the rate of formation of dicentric chromosomes after 2 Gy γ-irradiation was assessed, in comparison to non-irradiated control cells, indicating the extent of chromosomal instability after radiation exposure. The survival curve showed a curve-linear shape with a less pronounced shoulder (Figure 4A). The curve was fitted according to S = αD + βD^2^ [74], leading to a D_0_ of 1.2 Gy, a D_10_ of 4.7 Gy and a surviving fraction of 0.5 at 2 Gy (SF2). Thus, the cell line appears to be intermediately radiosensitive compared to 60 different tumor cell lines from different origins (solid and non-solid) investigated by Amundson *et al.* [75]. In their study, D_0_ values varied between 0.7 Gy and 7.25 Gy and the SF2 showed values between 0.04 and 0.95. The number of dicentric chromosomes was analyzed using a semi-automated soft- and hardware platform (Metafer4, version3.6.7). 71,621 chromosomes were evaluated for the non-irradiated control cells. In total, a spontaneous rate of dicentric chromosomes of 0.17 (±0.05) per 1,000 chromosomes was detected (Figure 4B). In comparison, 2 Gy γ-irradiation increased the dicentric rate in CAL 33 cells to 1.42 (±0.16) dicentrics per 1,000 chromosomes (57,649 chromosomes evaluated), which is 8.2-fold higher than the control. We also compared the dicentric rates of CAL 33 cells to spontaneous and radiation-induced dicentric chromosomes in normal human lymphocytes which are usually investigated in chromosome dosimetry studies [76]. The observed spontaneous rate of dicentric chromosomes for peripheral human lymphocytes with 0.012 (±0.012) dicentrics per 1,000 chromosomes (85,295 chromosomes analyzed) was lower by a factor of 14 compared to the detected spontaneous rate of CAL 33 cells. After irradiation, exposed lymphocytes showed an increased rate of dicentrics of 2.47 (±0.29) dicentric chromosomes per 1,000 chromosomes (28,391 chromosomes analyzed), *i.e.*, 1.7-fold higher than irradiated CAL 33 cells. Thus, lymphocytes are much more sensitive to radiation, which is in agreement with cell survival published by Kutlaca *et al.* [77]. These results lead to the following conclusions: (i) A pronounced radiation effect can be detected when analyzing the rate of dicentric chromosomes in CAL 33 cells after 2 Gy γ-irradiation; (ii) compared to normal human lymphocytes the spontaneous rate of dicentric chromosomes is significantly higher (*p* < 0.05; Fishers’ exact test) and (iii) the HNSCC cell line CAL 33 is less sensitive to ionizing radiation when compared to normal human lymphocytes. The resulting survival curve of CAL 33 HNSCC cells is in good concordance to published data [72] and several earlier studies already confirmed high radiation sensitivities for human lymphocytes [78,79,80]. The obtained data of CAL 33 cells can serve as a starting point for the determination of radiosensitivity of genetically engineered cell culture models based on this cell line.

## 3. Experimental Section

### 3.1. Cell Culture

CAL 33 cells were obtained from the “Deutsche Sammlung für Mikroorganismen und Zellkulturen” (DSMZ; No.: ACC447) and maintained in RPMI 1640 medium with L-Glutamine (PAA Laboratories, Cölbing, Germany) supplemented with 10% Fetal Bovine Serum (FBS; Sigma, Taufkirchen, Germany), 50 U/mL Penicillin and 50 µg/mL Streptomycin (Pen Strep, Gibco^®^ Invitrogen, Karlsruhe, Germany) in humidified incubators at 37 °C and 5% CO_2_. Subculturing of semi-confluent cultures was performed by using 0.25% Trypsin/EDTA (Gibco/Invitrogen, Karlsruhe, Germany) and cells were plated at densities between 10^4^ and 3 × 10^4^/cm^2^. Cells were mycoplasma-free as tested by the MycoAlert mycoplasma detection kit (Lonza, Verviers, Belgium). According to DSMZ, CAL 33 cells are negative for EBV, HBV, HCV, HIV and HTLV-/II and are hyperdiploid with 4% polyploidy and a unique DNA profile. 

### 3.2. Array CGH

Array CGH was performed as described previously by Bauer *et al.* [11]. Briefly, 450 ng of test DNA and 450 ng of sex-mismatched reference DNA (Promega, Madison, WI, USA) were labeled with Cy3-dCTP and Cy5-dCTP (PerkinElmer, Boston, MA, USA) respectively using a labeling kit (BioPrime, Invitrogen, Karlsruhe, Germany). Test and reference DNAs were cohybridized together with 135 µg cot-1 DNA on BAC-arrays of 1 MB resolution (CMR_Hs_1MB_Custom, Cambridge Genomic Services, University of Cambridge, Cambridge, UK) using a hybridization station (HS400, Tecan, Crailsheim, Germany). After 40 h and automated washing with 50% Formamide in 2 × SSC (pH = 7), 40% Formamide in 2 × SSC (pH = 7), 0.1% SDS in 2 × SSC and 0.1 × SSC, arrays were scanned (GenePix Personal 4100A) and fluorescence intensity ratios were measured using the array analysis software GenePix Pro 6.0 (both Axon Instruments, Molecular Devices, Sunnyvale, CA, USA). Further data analysis and visualization of CGH profiles were carried out as described by Unger *et al.* [10] and Bauer *et al.* [11] using the web-based array CGH evaluation platform CAPweb [81,82] and the array data visualization tool VAMP [83].

### 3.3. Metaphase Preparation

Chromosome preparations were needed for SKY and dicentric analysis as well as for testing BAC clone specificity for FISH. For metaphase spreads 2.5 × 10^5^ cells were grown in 4 mL media on a sterile glass slide positioned in a slide tray plate chamber (PAA, Pasching, Austria) and addition of 0.05 µg/mL Colcemid (Roche, Penzberg, Germany) overnight arrested cells in metaphase. After 24–32 h growth, the media was removed and the slide covered with 4 mL hypotonic KCl-solution (0.075 M). After incubation under hypotonic conditions for 20 min at 37 °C, 4 mL ice-cold fixative (methanol/glacial acetic acid, 3:1) were added followed by another incubation step for 20 min on ice. Subsequently the solutions were removed and replaced by another 4 mL of ice-cold fixative. After 20 min incubation on ice this last step was repeated. Finally, the slides were air dried perpendicularly under a laminar flow. 

### 3.4. Spektral Karyotyping (SKY)

SKY was performed as described previously by Zitzelsberger *et al.* [84]. Briefly, chromosome preparations were pretreated with RNase A (0.1 mg/mL in 2 × SSC) prior to hybridization. Chromosome denaturation was achieved by placing the slides in 70% formamide in 2 × SSC at 72 °C for 1–2 min. Subsequently, the slides were dehydrated in a 70%, 90% and 100% ethanol series and hybridized with a denatured SKY-probe mixture (SKYPaint^TM^ DNA Kit, Applied Spectral Imaging, Edingen-Neckarhausen, Germany). After hybridization (24 h), slides were washed using a rapid wash protocol: 0.5 × SSC for 5 min at 75 °C, 4 × SSC/0.1% Tween for 2 min and H_2_O_bidest_ for 2 min, both at room temperature. Probe detection was achieved using anti-digoxigenin (1:250; Roche, Penzberg, Germany), avidin-Cy-5 and avidin-Cy-5.5 antibodies (both 1:100; Biomol, Hamburg, Germany) according to the manufacturers’ protocols. Metaphase spreads were counterstained with 0.1% 4’,6-diamidino-2-phenylindole (DAPI) in antifade solution (Vectashield mounting medium; Vector Laboratories, Burlingame, CA). Chromosome aberrations were detectable by color junctions within affected chromosomes and a minimum of 15 metaphases were analyzed to determine a karyotype. Image acquisition was done using a SpectraCube system and analyses were accomplished using the SKYView imaging software (both Applied Spectral Imaging, Edingen-Neckarhausen, Germany). 

### 3.5. FISH

FISH analyses were performed using BAC clones from the 1 MB and 32 k Re-Array BAC Libraries (Wellcome Trust Sanger Institute, Hinxton, Cambridge, UK and BACPAC Resources Center, Children’s Hospital Oakland Research Institute, Oakland, CA, USA, respectively). Hybridizations of adequate BAC clones on metaphase spreads were used in order to define the cell lines’ karyotypes more precisely. BAC DNA was extracted according to standard procedures and labeled with digoxigenin and/or biotin by nick translation using commercially available nick translation kits (Roche, Penzberg, Germany). Metaphase slides were denatured in 70% formamide-2 × SSC for 0.5–1 min and dehydrated in an ethanol series (70, 90 and 100%). Hybridization was performed at 37 °C in a humid chamber for 48–72 h with labeled BAC probes (labeled BAC DNA precipitated with 2.5 volumes ethanol (100%) at −20 °C over night in presence of a 30-fold amount Cot1-DNA (1 mg/mL; Roche, Penzberg, Germany), 50-fold herring sperm DNA (11 mg/mL; Sigma-Aldrich, Taufkirchen, Germany) and 2% glycogen (20 mg/mL; Roche, Penzberg, Germany)) that had been denatured at 76 °C prior to use. Slide washing as well as detection of signals was performed as described previously by Unger *et al.* [85]. As a variation, DTAF conjugated streptavidin (Jackson ImmunoResearch, West Grove, PA, USA) was used instead of streptavidin-FITC for detection of biotinylated probes. Evaluation of prepared slides was done using a fluorescence microscope equipped with a charge-coupled device (CCD-) camera and the ISIS software (Metasystems, Altlußheim, Germany).

### 3.6. Dicentric Chromosomes

To analyze the frequency of dicentric chromosomes after γ-irradiation, cells were grown to complete confluency assuming that most of the cells enter G0 phase in response to a lack of space, growth factors and nutrients. Cells then were trypsinized, counted, divided up in two parts (0 Gy = control and 2 Gy), and irradiated *in vitro* using a Cesium (^137^Cs) source (HWM D-2000, dose rate 0,54 Gy/min, Wälischmiller Engineering, Markdorf, Germany). Control cells (0 Gy) were mock treated. For preparations of metaphase spreads, irradiated and control cells were grown on sterile glass slides and treated as described in the metaphase preparation section above. Additionally, 2 µM 5-bromo-2’-deoxyuridine (BrdU) per mL medium were added in order to distinguish metaphase cells in the first replication cycle (M1) from cells that are already running through the second replication cycle (M2) after irradiation. The differentiation between M1 and M2 metaphases was achieved by the fluorescence plus giemsa (FPG) staining, first described by Perry and Wolff [86]. To begin with, metaphase preparations were stained for 12 min in 1 µg/mL bisbenzimide (Serva, Heidelberg, Germany). After 10 min UV-irradiation at 60 °C metaphase preparations were stained in 10% giemsa solution (Merck, Darmstadt, Germany) in PBS and sealed with quick hardening mounting medium (Eukitt^®^, Fluka Analytical, Sigma-Aldrich, Taufkirchen, Germany). Evaluation of dicentric chromosomes was achieved by the image analysis module DC-Score of the software platform Metafer 4 version 3.6.7 (Metasystems, Altlußheim, Germany). In detail, microscope slides were automatically screened for metaphase figures by the software in combination with a microscope system (Axioplan2 imaging, Zeiss, Oberkochen, Germany) at a low magnification (×10) and X-Y positions were stored. In a second screening cycle, every metaphase spread was captured at a higher magnification (×63). Unsuitable images were then rejected interactively and the remaining metaphases were analyzed automatically for the existence of dicentric or multicentric chromosomes. Interactive re-analysis either confirmed valid dicentrics or removed false positives. Results were calculated as number of dicentrics per 1,000 chromosomes.

### 3.7. Cell Survival Curves

Clonogenic cell survival after different doses of γ-irradiation was investigated by determining the fraction of surviving cells via standard colony forming assay (CFA). Exponential cell cultures were exposed to increasing doses of γ-irradiation (0, 1, 2, 3, 4, and 6 Gy) using a Cesium (^137^Cs) source (HWM D-2000, dose rate 0.54 Gy/min, Wälischmiller Engineering, Markdorf, Germany), plated in a defined number and incubated for 2–3 weeks at 37 °C and 5% CO_2_ until macroscopically visible colonies formed. 50 Gy irradiated non-multiplying feeder cells were used in order to improve the plating efficiency of investigated CAL 33 cells [87]. For evaluation, colonies were fixed in methanol and stained in 10% giemsa solution (both Merck, Darmstadt, Germany). Only colonies >50 cells were taken into consideration for evaluation using a binocular microscope (Olympus, Hamburg, Germany). The survival fraction was calculated by dividing the number of colonies counted by the number of cells plated divided by the fraction of the plating efficiency of the non-irradiated control. The surviving fractions were then plotted on a logarithmical scale *versus* dose in Gy on a linear scale to generate survival curves. Three independent experiments were carried out and the resulting data were fitted according S = αD + βD^2^ [74].

### 3.8. Transfection of CAL 33

CAL 33 cells were stably transfected with a linearized FANCA-overexpressing vector (pcDNA3.1(+)/FANCA) using the transfection reagent Polyfect^®^ (Qiagen, Hilden, Germany) according to the manufacturers’ protocol in 6-well plates. 48 h after transfection selection of stably transfected cells was achieved by addition of 200 µg/mL Geneticin (G418; Sigma- Aldrich, Taufkirchen, Germany). Cells were cultivated as described above in the cell culture section and trypsinized at about 80% confluency. Cell cloning of transfected cells was carried out by dilution in 96-well plates. Each well was checked regularly for clone growth and wells containing a single colony were labeled. Clones were further cultivated and stocks were frozen in liquid nitrogen.

## 4. Conclusions

Head and neck cancer cell line CAL 33 is an appropriate cell culture model system for functional and mechanistic studies, since it mirrors well the cytogenetic status of primary HNSCC. CAL 33 cells are of epithelial origin and display chromosomal rearrangements and copy number changes known from studies on primary HNSCC. However, karyotypic and clonal evolution during cell culturing has to be considered, as this can influence and modify the phenotype and behavior of cells. Also genetic engineering (*i.e.*, transfection experiments) can lead to karyotypic changes that might affect functional studies and their results. Complementary cytogenetic approaches like SKY, array CGH and FISH are suitable tools to observe alterations in cell culture systems, and should be utilized for the careful routine analysis of cells.
